# Anti-IgLON5 Disease – The Current State of Knowledge and Further Perspectives

**DOI:** 10.3389/fimmu.2022.852215

**Published:** 2022-03-01

**Authors:** Natalia Madetko, Weronika Marzec, Agata Kowalska, Dominika Przewodowska, Piotr Alster, Dariusz Koziorowski

**Affiliations:** ^1^ Department of Neurology, Medical University of Warsaw, Warsaw, Poland; ^2^ Students’ Scientific Circle of the Department of Neurology, Medical University of Warsaw, Warsaw, Poland

**Keywords:** IgLON5 disease, IgLON5 antibodies, autoimmune disease, neurodegeneration, neuroinflammation

## Abstract

Anti-IgLON5 disease is a relatively new neurological entity with the first cases reported in 2014. So far, less than 70 articles on this topic have been published. Due to its unspecific symptomatology, diverse progression, novelty and ambiguous character, it remains a difficulty for both clinical practitioners and scientists. The aim of this review is to summarize the current knowledge concerning anti-IgLON5 disease; mechanisms underlying its cause, symptomatology, clinical progression, differential diagnosis and treatment, which could be helpful in clinical practice and future research.

## Introduction – IgLON-5 - Physiological Role, Pathogenetic Mechanism and Epidemiology

The IgLON family consists of five genes: Lsamp, Ntm, Opcml, Negr1, and Iglon5. It is well known for being involved in the process of neuronal adhesion, neurogenesis and neuroplasticity and is strictly regulated by metalloproteinase activity on the surface of mature cortical neurons (1–3). IgLON genes can also regulate the level of renal perfusion due to glomerular afferent and efferent innervation and IgG anti-IgLON5 reactivity against renal glomeruli (3, 4). The molecular structure of the IgLON5 protein can induce the production of specific antibodies in some cases, whose presence is crucial for anti-IgLON5 disease diagnosis, occurring with nonspecific symptoms like breathlessness during sleep, cognitive deterioration, gait disturbance and dysregulation of autonomic central nervous system (CNS) activity (5). The first cases of patients presenting i.a. severe air-flow disturbance in the respiratory tract and sleep apnea concurrently with the presence of anti-IgLON5 in cerebrospinal fluid (CSF) or serum were described in 2014 (6, 7). Furthermore, IgLON5 is suspected of maintaining blood-brain barrier (BBB) integrity and – as previously mentioned – is involved in processes related to neuroplasticity and neurogenesis (8–10). Characteristic of anti-IgLON5 disease is not only the presence of specific anti-IgLON5 antibodies, but also deposits of hyperphosphorylated tau located mainly in the tegmentum of the brainstem and hypothalamus (11).

It has not been fully established whether intense inflammatory response mediated by antibody activity leads to neurodegeneration or if the ongoing tau pathological accumulation exacerbates the immunological response (5). Therefore, the exact nature of the disease remains unclear.

**Figure 1:**
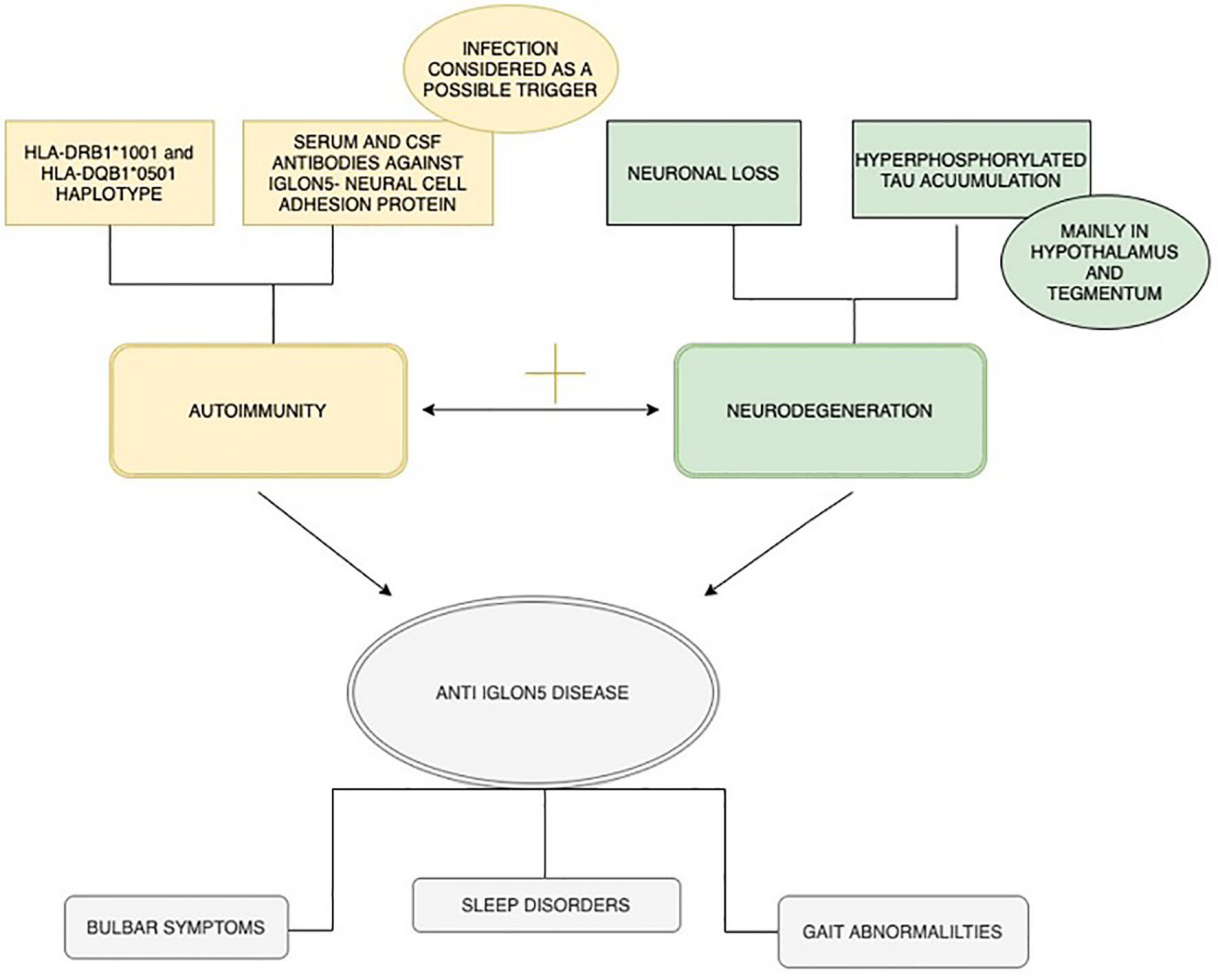
Possible heterogenous mechanism of anti-IgLON5 disease.

Interestingly, whether or not it is confirmed that the disease is combined with intensive gliosis and neuronal loss, the presence of immune cells engaged in the inflammatory process remains a matter of dispute (11). Possible heterogenous pathogenetic mechanism of anti-IgLON5 disease is summarized in **Figure 1**.

Prevalence of anti-IgLON5 antibodies is estimated at 12 out of 150 000 patients per year, although it is believed to be higher due to numerous cases being incorrectly diagnosed and reported (5, 12). Most commonly, diagnosis is made among elderly patients, with no sex predominance, although the disease onset is designing and may occur between the ages of 45 and 70 (5, 13). The literature points out that anti-IgLON5 antibodies found in serum or CSF are the necessary element of a definite diagnosis (11). A strong association between this disease and the presence of HLA-DRB1*10:01 and HLA-DQB1*05:01 alleles (Human Leukocyte Antigens) may be evidence for the theory of genetic predisposition to autoimmune disease development, but infections are also considered as a potential disease trigger (1, 6, 14).. IgLON5-antibodies can co-exist with anti-γ-aminobutyric acid B (GABA-B)-receptor molecules, associated with small-cell lung tumor development and seizures (15, 16).

## Discussion

### Anti-IgLON5 – Clinical Overlaps and Impact on Other Disorders

*In vitro* investigations performed on neuronal cells revealed that anti-IgLON5 disease leads concurrently to a decrease of the neuronal spikes ratio and enhanced accumulation of tau protein. Distinct localization of tau-deposits compared to that found in another neurodegenerative diseases, e.g. Progressive Supranuclear Palsy (PSP) and Corticobasal Syndrome (CBS), could indicate that anti-IgLON5 is a novel tauopathy (17). Neuropathological investigations have shown that hyperphosphorylated tau has a propensity to gather in structures located in the basal part of the brain, including the hypothalamus, the tegmentum and periaqueductal gray matter (5). The correlation between the amount of aggregates and symptom severity needs to be investigated further.

The wide spectrum of possible clinical symptoms requires an intensive differential diagnostic process of anti-IgLON5 disease versus other syndromes and verification of whether they can develop simultaneously. Sleep abnormalities mimicking anti-IgLON5 can be observed in the MM2-thalamic subtype of sporadic Creutzfeldt-Jakob disease with Lewy bodies, whereas patients with gaze palsy and postural instability – tending to be diagnosed with probable PSP – can also develop these symptoms as a result of anti-IgLON5 disease (18–20). Furthermore, recent literature indicates a correlation between anti-IgLON5 disease and the occurrence of schizophrenia and renal neoplasia (9, 21). Since tumor-suppression seems to be one of the IgLON family functions, genetic mutations leading to their defective activity have a high likelihood of resulting in an increased risk of cancer development, mostly in the urogenital and reproductive systems (2, 22). Expression of the IgLON5 gene correlates with unfavorable renal cancer survival expectancy, but it is still thought that paraneoplastic activity of IgLON5 expression is rare (3, 23). A unique case of a pediatric patient with Langerhans cell histiocytosis (LCH) and co-existing IgLON5 disease has also been described (24). A child had rapidly developed increased muscle tone during chemotherapy, presumably confirming the theory of IgLON5 having a role in maintaining an equable muscular microenvironment and its regeneration (24, 25).

In as much as anti-IgLON5 is described in the literature as a neuroimmune disease, it seems that the mechanism of indivertible internalization of the IgLON5 cluster, proceeded by auto-antibodies, could be a reason for a poor immunotherapy response in a group of treated patients (17, 26, 27). Although in many cases treatment based on various immunosuppressive blends (consisting of corticosteroids, rituximab, cyclophosphamide, azathioprine and mmycophenolate mofetil) in combination with intravenous immunoglobulin (IVIG) have positive therapeutic results, it is worth mentioning that anti-IgLON5 disease is a potentially fatal condition, which can lead to sudden death due to laryngospasm or aspiration (28).

Prompt and precise diagnosis focused on detection of specific antibodies in patients with common clinical symptoms like sleeping abnormalities, postural instability or memory impairment seems to play a crucial role in therapy, in order to offer the most relevant treatment as soon as possible (29).

### Clinical Manifestations of Anti-IgLON5 Antibody-Related Syndrome

Anti-IgLON5 disease, a progressive disorder which combines autoimmunization with neurodegeneration, may have a very heterogenous manifestation. The core of the clinical presentation consists of sleep disorder, bulbar symptoms and gait abnormality followed by cognitive dysfunction (12, 30, 31). Other common symptoms include oculomotor abnormalities such as vertical/horizontal gaze palsy, nystagmus or ptosis (28, 32). Dysautonomia presenting with nycturia, urinary urgency, anhidrosis or constipation was also reported in these patients. Alongside the aforementioned, there were also cerebellar symptoms and indications from the peripheral nervous system such as muscle weakness, stiffness and fasciculations (12, 28). Reported movement disorders can be hyperkinetic or hypokinetic, and comprise chorea, bradykinesia, tremor, dystonia, myokymia, myorhythmia. They are very often seen in craniofacial areas, but can be present in any body region and can even be generalized (1, 28, 33). Apart from cognitive decline, there have also been other neuropsychiatric symptoms such as hallucinations or memory loss (13, 28, 29). The course of the disease is chronic in most cases as symptoms usually develop over years; however, it can also be subacute or even acute (weeks) (34). Recently published articles analyzed the primary reason why patients suffering from this condition were seeking a neurological consultation (1, 35). Results indicate that most of the patients affected with anti-IgLON5, experience sleep disorders to an extent that forces them to look for medical help. Although sleep disorder, bulbar symptoms and gait abnormality form the main symptoms of anti-IgLON5 disease, they can differ in severity and onset, making various combinations with other clinical features present. In previous studies there have been a few clinical phenotypes of the disease identified on this basis,: 1) sleep disorder predominance, 2) a bulbar syndrome, 3) progressive supranuclear palsy-like syndrome, 4) cognitive disorder with or without chorea (1). Recently, there have also been a few cases of patients with a phenotype resembling motor neuron disease reported. They predominantly suffered from dysphagia, limb weakness, spasticity, tongue fasciculations and intrinsic hand muscle atrophy (3, 6, 36). Stiff-person-like syndrome has also been identified among patients with anti-IgLON5 disease in recently published studies. Muscle rigidity and hyperekplexia are the most characteristic features of this phenotype (3). It is without doubt that anti-IgLON5 disease can have a very wide range of symptoms and that this spectrum will probably be broadening in line with the latest case reports and cross-sectional studies. Taking into consideration the multiplicity of the clinical manifestations and the variable progression of the disease, there is no doubt that making a correct diagnosis can be brain-busting in clinical practice.

### Sleep Disorders

Sleep abnormalities, which are very common among patients with anti-IgLON5 disease, can be variously characterized. Some of them are hard for the patient himself to notice and are reported during medical interview by the bedpartner (1). Sometimes patients tend to underestimate the importance of these kinds of symptoms, which can lead to delayed diagnosis. Sleep abnormalities connected with this entity include insomnia affecting sleep onset and fragmented sleep followed by excessive daytime sleepiness, vocalizations, abnormal limb movements, jerks, snoring, sleep apnea and stridor during sleep (1, 36) Polysomnography with synchronized audiovisual recording is crucial during anti-IgLON5 diagnosis. Sleep pattern characteristic for the disease were described as “undifferentiated-NREM (UN-NREM) and poorly-structured N2 (P-SN2)” (37). Diffuse irregular theta waves with increased EMG activity and vocalization occur in UN-NREM phase whereas in P-SN2 “occasional K-complexes or sleep spindles associated with EMG activation” and motor behavior are observed (37). It appears that anti-IgLON5 has a distinctive sleep disorder pattern which is unique to this disease. A recently completed study showed that parasomnias and breathing disorders are present during both NREM and REM sleep phase. Anti-IgLON5 sleep disorder can be characterized by abnormal NREM sleep initiation, rapid eye movement sleep behavior disorder (RBD), motor activation and breathing problems such as stridor and apnea (38, 39).

### Gait Abnormality

Gait abnormality is very often the reason why patients, subsequently diagnosed with this condition, visit a neurologist for the first time (1). Usually they experience difficulties walking independently with feelings of imbalance and retro/lateropulsion. Some of them tend to have gait freezing. Gait problems can be mild, moderate or severe with frequent falls among patients with pronounced postural deficiencies (28). These symptoms can be attributed to various causes, including cerebellar ataxia, muscle stiffness and dystonia. All of the above mentioned are linked with anti-IgLON5 disease (1). A recently published case report revealed another possible feature responsible for gait abnormality in anti-IgLON5 disease – bilateral vestibulopathy, which appears to be another sign of peripheral neuropathy in this condition (40).

### Bulbar Syndrome

Bulbar syndrome forms the third most common set of symptoms characteristic for anti-IgLON5 disease (1). The most frequently reported one is dysphagia, which very often leads to substantial weight loss (28). In some patients, it can even be the initial sign of the disease (41). Dysarthria, presenting with hoarseness and vocal cord palsy, laryngospasm and central hypoventilation is also reported (28, 42).

### Progressive Supranuclear Palsy-Like Syndrome

Progressive Supranuclear Palsy is a neurodegenerative disease and one of the Atypical Parkinsonian Syndromes. Its most characteristic clinical features comprise oculomotor abnormalities and posture instability followed by akinesia and cognitive dysfunction (20). Sometimes patients with anti-IgLON5 disease present a set of symptoms closely resembling those characteristic for Progressive Supranuclear Palsy (PSP) or even meeting the diagnostic criteria for this condition (9). The fact that typical sleep abnormalities occur among patients with anti-IgLON5 disease alongside PSP-like symptoms might be helpful during the process of differential diagnosis (43). What’s more, a recent study showed that oculomotor abnormalities tend to differ between the two conditions and assessing them may be a useful tool in making the right diagnosis. Supranuclear gaze palsy appears to be much more characteristic for PSP. Saccade velocity and accuracy evaluated in Video-Oculography differs as well (43).

### Cognitive Impairment With/Without Chorea

Cognitive decline is a common clinical manifestation of anti-IgLON5 disease and can even lead to dementia in severe cases. It often occurs alongside chorea (1). Sometimes it even appears as a main symptom of the condition causing deterioration to the patient’s quality of life and self-management abilities. Patients can present various cognitive defects including verbal and visual memory, verbal fluency, or selective attention (44).

## Diagnostic of Anti-IgLON5

### Antibody-Related Syndrome

When a patient presents with heterogeneous neurological symptoms including distinctive sleep disorders often accompanied by bulbar symptoms, gait instability or cognitive deterioration, anti-IgLON5 disease should always be suspected. Early diagnosis and implementation of immunotherapy has a positive impact on the course of the disease and can prevent possible complications. Therefore, it is crucial for a physician to carefully assess the whole clinical presentation (1). The most important examinations to confirm or rule out this condition are testing Cerebrospinal Fluid (CSF) and serum for antibodies against IgLON5 through immunohistochemistry. They can be present in both of the fluids mentioned above and are characteristic for this entity (1, 27, 28). Analyses of IgG subclasses in serum and CSF by cell-based assay and flow cytometry show the presence of IgG4 and IgG1 with a substantial predominance of IgG4 antibodies (1, 27, 28). There is also a significant association between certain haplotypes and the presence of the disease suggesting a particular genetic predisposition. Studies revealed that human leukocyte antigen (HLA) HLA-DRB1*10:01, which strongly segregates with DQB1*05:01, is strongly correlated with the presence of anti-IgLON5 antibodies and is 36 times more frequent among patients with this condition compared to the general population (1, 45). Therefore, HLA genotyping of a patient suspected of suffering from this condition can be a helpful tool in making the correct, definite diagnosis. Anti-IgLON5 disease is also associated with certain neuropathological findings which were detected during postmortem examination of brain samples (7, 11). Hyperphosphorylated tau deposits are most characteristic, with the presence of both isoforms of this protein (three-repeat and four-repeat), especially in the tegmentum of the brainstem and the hypothalamus, alongside neuronal loss. Based on these neuropathological features, together with the presence of anti-IgLON5 antibodies, certain haplotypes and particular clinical manifestations, diagnostic criteria with different levels of certainty have been proposed. A recommended minimal sampling protocol for the diagnosis of anti-IgLON5 has also been established (11). Definite diagnosis can be made if distinctive neuropathological findings are accompanied by detection of anti-IgLON5 antibodies in CSF or serum. Probable diagnosis can be established when the status of antibodies is unknown, but only if neuropathological findings coexist with a characteristic clinical history or the presence of the HLA-DRB1*10:01 HLA- DQB1*05:01 haplotype. When the above mentioned neuropathological features are present, but there is no information about any characteristic clinical symptoms or immunological status, anti-IgLON5 disease can be considered only as a possibility. Neuropathological diagnostic criteria are summarized in **Table 1**.

**Table 1:** Anti-IgLON5 neuropathological diagnostic criteria.

Level of certainty	Neuropathological findings	CSF/serum Anti-IgLON5 antibodies	Clinical history	HLA-DRB1*10:01 HLA- DQB1*05:01
Definite	+	+	+/-	+/-
Probable	+	No information	+/-	+/-
Possible	+	No information	No information	No information

Magnetic resonance (MRI) is not particularly helpful in making a diagnosis because of its indeterminate and nonspecific findings in most patients. However, sometimes patients can present mild brainstem and cerebellar atrophy in brain MRI images (1, 28, 46). CSF examination, electroencephalography (EEG) or electromyography (EMG) also show no distinctive changes (1). Due to the fact that sleep disorder is one of the most characteristic abnormalities in anti-IgLON5 disease, video polysomnography (VPSG) should be performed in any suspected case of this condition, not only on patients who complain of sleep problems. Sometimes certain sleep disorder symptoms such as abnormal movements during sleep or obstructive sleep apnea are difficult for the patients to notice themselves. Therefore, VPSG can be a relevant tool in dealing with a broadening spectrum of clinical symptoms and guiding the physicians in the right direction in terms of providing an accurate diagnosis and the correct treatment. Characteristic findings for anti-IgLON5 disease in VPSG examination are abnormal NREM sleep initiation, rapid eye movement sleep behavior disorder (RBD), motor activation and breathing problems such as stridor and apnea (38, 39).

In conclusion, the right diagnostic approach should comprise reliable clinical assessment, testing CSF and serum for anti-IgLON5 antibodies, HLA genotyping and performing VPSG. Brain MRI, CSF examination, EEG and EMG can be helpful only in terms of excluding other possible conditions.

### Differential Diagnosis of Anti-IgLON5 Antibody-Related Syndrome

Due to the fact that anti-IgLON5 disease has only been described relatively recently it still remains unknown to many physicians, including neurologists. Differential diagnosis varies depending on the predominant symptoms and the clinical subtype of the disease. The most common clinical manifestation in the form of sleep disorder may be misdiagnosed as isolated obstructive sleep apnea syndrome, status dissociatus, agrypnia excitata, idiopathic RBD, conventional NREM parasomnias or other autoimmune encephalitis (7, 13, 35, 39). Status dissociatus can be seen in narcolepsy, neurodegenerative disorders like advanced dementia with Lewy bodies or multiple system atrophy, brainstem lesions or as a side effect of treatment with multiple psychoactive drugs. Agrypnia excitata, a more severe condition, can occur in fatal familial insomnia, Morvan syndrome or delirium tremens (34, 39, 47). Therefore, in each of the aforementioned cases, anti-IgLON5 disease should also be considered.

Patients with bulbar dysfunction can resemble myasthenia gravis, including the myasthenic crisis, or motor neuron disease (36, 41, 48). In patients with severe gait instability and falls, PSP should be considered, especially when there are additional oculomotor abnormalities, or MSA, particularly when dysautonomia also occurs (1, 19, 32). Atypical dementia may be suspected if cognitive problems predominate (35). In cases where, in addition to cognitive impairment, chorea is//present, Huntington’s disease can be misdiagnosed (49, 50). Myorrhythmia with sleep disorder and hypothalamic dysfunction might resemble Whipple disease (3, 51). If patients present symptoms of nervous system hyperexcitability, stiff-person-like syndrome is often considered (12, 35). Another autoimmune disorder that should be taken into consideration in differential diagnosis is celiac disease. Although the disease is mainly connected with gastrointestinal symptoms, about 10% of patients with celiac disease develop diverse neurological symptoms among which i.a. dementia, chorea, leukoencephalopathy, cerebellar ataxia and myoclonus can be mentioned. The exact mechanism of this association remains unclear, however autoimmune reaction is described as one of the possibilities, what is supported by the presence of antineuronal antibodies in sera of patients with celiac disease with neurologic manifestation. Study conducted by Cervio et al. (52) indicated that sera with antineuronal IgG and IgA antibodies obtained from patients with celiac disease and neurological involvement cause mitochondrial-dependent apoptosis in human neuroblastoma cell line. Another papers indicated significant correlation between presence of anti-ganglioside antibodies (anti-GM1, anti-GD1b, anti-GQ1b) in the serum of celiac patients and incidence of neurological manifestations (53, 54). Therefore, anti-ganglioside antibodies may be treated as a marker of neurological involvement in celiac disease and prove its autoimmune etiology.

Other diseases that need to be taken into consideration in differential diagnosis include infectious encephalitis and autoimmune epilepsy (3, 14, 55, 56).

In conclusion, anti-IgLON5 disease can mimic many different conditions, so whenever a patient does not fulfill the diagnostic criteria for the above-mentioned disorders or has significant sleep disturbance, IgLON5 antibodies should be tested.

### Treatment of Anti-IgLON5 Antibody-Related Syndrome

The effectiveness of immunotherapy is still debated, but due to the lack of other treatment options, it seems to be the first line of therapy for anit-IgLON5 disease (50). An insignificant response to immunotherapy was initially described in case reports (1, 7, 12, 41), but the response increased over time, which could be explained by the diagnosis of the disease in patients with atypical clinical manifestations (3, 28, 46, 48, 50, 55, 57). In one of the largest systematic reviews, 20 out of 46 patients (43.4%) responded to immunotherapy, and presence of response to last follow-up was recorded in 15 of them (58). The most frequently used treatment methods are cycles of corticosteroids i.v., immunoglobulins i.v., TPE (therapeutic plasma exchange), rituximab, cyclophosphamide, azathioprine and mycophenolate mofetil (28, 55). Combination therapy and the use of second-line treatments seem to be more effective than monotherapy and provide a sustained response more often (28, 58, 59). Factors that may increase the effectiveness of the treatment include cognitive impairment and non-classical phenotypes, the presence of HLA-DQB1*05:01 without the HLA-DRB1*10:01, IgG1 subtype and cerebral spinal fluid inflammation, which might indicate the active phase of the disease and therefore enable the efficacy of immunotherapy (48, 55, 58). One study reported that resolution of neurological symptoms may be associated with a decrease in IgLON5 antibodies (60). Usually the diagnosis was made when the disease was moderate to severe (13). Some studies have emphasized the importance of early initiation of immunotherapy (48), but others have not shown any correlation between disease duration and response to immunotherapy (28, 58). Nevertheless, one of the studies emphasized that in many cases there was no data concerning disease duration before starting treatment, hence it seems that immunotherapy should be started as early as possible (58).

Neuroimmune characteristics of anti-IgLON5 disease may be a clue in therapy selection. As the disease is caused by the presence of immunoglobulins G4 (IgG4) subclass, which is unable to activate complement and complement-mediated immune response, rather poor improvement after IVIG monotherapy can be expected (61). Instead, rituximab or novel anti-CD19/20 monoclonals like inebilizumab or obexelimab (currently in phase-3 clinical tirals) should be taken into consideration (61). Therefore, based on the immunological characteristics of the disease, the Authors suggest to use combination therapy with rituximab as a comprehensive approach ensuring the best possible results.

Positive airway pressure treatments (CPAP, BiLevel therapy) can be used in patients with obstructive sleep apnea (62). They improve respiratory symptoms, but have no influence on abnormal sleep behavior and movements (39). In case of respiratory failure, for example in the course of paralysis of the vocal cords, an intubation or tracheotomy might be necessary (34). Treatment with dopaminergic, neuroleptic, antiepileptic and GABAergic drugs does not usually alleviate the symptoms. The use of anticholinergic drugs may even worsen the symptomatology (34). Therapeutic approach is summarized in **Table 2**.

**Table 2:** The most commonly used drugs in monotherapy and combination therapy for anit-IgLON5 disease.

**Monotherapy**	corticosteroids i.v.
	IVIg
	plasmapheresis
	rituximab
**Combination therapy**	IVIg + corticosteroids
	IVIg + plasmapheresis
	corticosteroids + azathioprine
	IVIg + mycophenolate mofetil
	IVIg + cyclophosphamide
	**IVIg + plasmapheresis + rituximab – recommended**

The bold values were implemented to highlight the authors' recommendations considering treatment.

The prognosis is usually poor with high mortality. The most common causes of death include central hypoventilation, sudden death of unknown reason while sleeping or awake, bradycardia or as a consequence of aspiration pneumonia due to dysphagia (13). There are a few cases where patients improved without immunotherapy, including patients with infection preceding symptoms, whose titer of serum anti-IgLON5 antibodies decreased with antiviral drugs (14, 55). This may increase the plausibility of a parainfectious cause of anti-IgLON5 disease in some patients and its resolution when viral infection is treated.

It seems that aggressive and combined immunotherapy appears to be crucial for positive outcomes and should be initiated as soon as possible. However, due to the lack of large studies that would clearly indicate the advantage of one drug over another, or determine the best time to start treatment, more research in the field based on larger groups is required.

## Conclusion

Anti-IgLON5 disease is a very broad entity with multiple clinical manifestations and unclear pathophysiology. The disease course can have diverse dynamics and multiple overlaps with other disorders. Taking into consideration its novelty and ambiguous character, currently it would be more relevant to describe it as an IgLON5 antibody-related syndrome.

Anti-IgLON5 related syndrome provides another argument in the discussion concerning the role and possible correlation between neuroinflammation and neurodegeneration. Research concerning exact mechanisms of the development of the disease could lead to an explanation, whether neuroinflammation precedes neurodegeneration or activation of the immune system in CNS is a response to the accumulation of pathologic proteins. It is already known that an increased inflammatory response exacerbates the progress of the disease in PD and atypical parkinsonian syndromes (63). The role of neuroinflammatory reaction in the development and progression of neurodegenerative diseases should be extensively examined as it might be the key to its causal treatment in the future.

Anti-IgLON5 is a clinically non specific disease with an indicating point based on immunological assessment. This, due to the overlaps with entities lacking feasible *in vivo* assessment as PSP may be interpreted as a beneficial feature of examination. In PSP basically the diagnosis during the *in vivo* assessment is affected by significant overlaps with PD in early stages and other atypical parkinsonisms in the more advanced periods. Physicians also experience a characteristic feature of the assessment of tauopathic atypical parkinsonisms, which is their limited correlation of clinical manifestation and underlying pathology. This is partly evitable in anti-IgLON5 - the antibody-related disorder, since its discovery has not been associated with any clinical method of comparable specificity as antibody testing. It seems that perspectives associated with IgLON5 are generally related to evolving treatment and *in vivo* clinical examination, however further exploration of the pathogenesis will facilitate moving forward in both grounds.

## Author Contributions

NM - study concept, writing, data analysis, and final acceptance. WM, AK, and DP - writing, data collection, and final acceptance. PA – writing and final acceptance. DK – supervision and final acceptance. All authors contributed to the article and approved the submitted version.

## Conflict of Interest

The authors declare that the research was conducted in the absence of any commercial or financial relationships that could be construed as a potential conflict of interest.

## Publisher’s Note

All claims expressed in this article are solely those of the authors and do not necessarily represent those of their affiliated organizations, or those of the publisher, the editors and the reviewers. Any product that may be evaluated in this article, or claim that may be made by its manufacturer, is not guaranteed or endorsed by the publisher.
